# Modeling the *C9ORF72* repeat expansion mutation using human induced pluripotent stem cells

**DOI:** 10.1111/bpa.12520

**Published:** 2017-06-05

**Authors:** Bhuvaneish T. Selvaraj, Matthew R. Livesey, Siddharthan Chandran

**Affiliations:** ^1^ MRC Centre for Regenerative Medicine University of Edinburgh Edinburgh EH16 4UU UK; ^2^ Euan MacDonald Centre for MND Research University of Edinburgh Edinburgh EH16 4SB UK; ^3^ Centre for Clinical Brain Sciences University of Edinburgh Edinburgh EH16 4SB UK; ^4^ Centre for Integrative Physiology University of Edinburgh EH8 9XD UK; ^5^ Centre for Brain Development and Repair inStem Bangalore 560065 Karnataka India

## Abstract

*C9ORF72* repeat expansion is the most frequent causal genetic mutation giving rise to amyotrophic lateral sclerosis (ALS) and fronto‐temporal dementia (FTD). The relatively recent discovery of the *C9ORF72* repeat expansion in 2011 and the complexity of the mutation have meant that animal models that successfully recapitulate human *C9ORF72* repeat expansion‐mediated disease are only now emerging. Concurrent advances in the use of patient‐derived induced pluripotent stem cells (iPSCs) to model aspects of neurological disease offers an additional approach for the study of *C9ORF72* mutation. This review focuses on the opportunities of human *C9ORF72* iPSC platforms to model pathological aspects of disease and how findings compare with other existing models of disease and post mortem data.

## Introduction

Expansion of a GGGGCC (G_4_C_2_) intronic hexanucleotide in the *C9ORF72* (chromosome 9, open reading frame 72) gene is the most common cause of familial ALS and accounts for ∼10% of sporadic ALS 1, 59, 68. The *C9ORF72* repeat expansion (*C9ORF72^RE^*) is also the most frequent underlying genetic cause of FTD 12, 59 and approximately 50% of ALS patients co‐present with FTD 57. Typically, the number of repeats in healthy individuals is lower than 30 and of several hundred repeats in affected individuals. Cytoplasmic TDP‐43 inclusions and C9ORF72^RE^ RNA foci are major histopathological features of both ALS and FTD 12, 45, 52. The pathogenicity of the *C9ORF72^RE^* mutation may act through two potential mechanisms; 1) a loss of function generated through haploinsufficiency of the *C9ORF72* gene, and 2) a gain of function mediated through transcribed and/or translated elements of the mutant intronic repeat expansion 74.

A range of animal and cellular models have been developed to understand the cellular perturbations generated by the *C9ORF72^RE^*. Valuable insights into *C9ORF72^RE^*‐mediated disease have been determined using animal models, which include *C. elegans*, zebrafish, drosophila and mice; collectively these reveal the complexity of potential disease mechanism (see 46 for a review). Indeed, the absence of TDP‐43 inclusions, RNA foci and neurodegeneration in *C9orf72* knock out mice 31 have demonstrated that loss of function is insufficient to generate disease though may contribute to an inflammatory component of the disease 55, 71. In contrast, *C9ORF72^RE^* mouse models that support a gain of function mechanism and successfully exhibit neurodegeneration with pathological hallmarks of *C9ORF72^RE^*‐mediated disease have only recently been developed 7, 38. Such genetically engineered models that follow past strategies that generated only partial recapitulation of the *C9ORF72^RE^* phenotype will undoubtedly generate important future insights. Animal models are reviewed elsewhere 46, 74. Conversely, iPSC technology now allows the derivation of cell‐type specific and regionally‐defined material that harbors the patient genetic background. This also allows to recapitulate the disease in physiological context wherein the mutation is expressed in physiological level. Concurrently, recent development of CRISPR/Cas9 mediated genome editing has paved a way to generate isogenic iPSC lines. This has allowed to study the role of mutation in a genetically defined manner and overcome the challenges of genetic variability between different iPSC lines 61. In this regard multiple studies on a range of *C9ORF72^RE^* patient iPSC‐derived cell types including motor neurons, cortical neurons, astrocytes and oligodendrocytes have been already performed. Beyond recapitulating major pathological signatures of disease, iPSC‐based platforms have yielded novel biochemical and physiological information previously unknown in the context of *C9ORF72^RE^*. In this review we will highlight how this technology has contributed to our understanding of *C9ORF72^RE^*‐mediated disease.

### C9ORF72 protein and loss of function hypothesis

The *C9ORF72* gene generates three different mRNA transcript variants (V1, V2, V3). Transcript variant 1 and 2 contain the repeat expansion mutation in the first intron therefore pre‐mRNA form V1 and V2 contains the G_4_C_2_ repeats, whereas transcriptional start site for variant 2 is after the repeat expansion mutation. Transcript V1 encodes for a short isoform of C9ORF72 protein, whereas transcript V2 and V3 encode for the long isoform of C9ORF72 protein. Although the structure of C9ORF72 protein is not yet elucidated, it is predicted to be related to differentially expressed in normal and neoplasia (DENN) protein which functions as a GDP/GTP exchange factor (GEF) in activating RAB GTPases 36. Localization studies in human post‐mortem tissues suggested that C9ORF72 short isoform is localized in the nuclear membrane interacting with components of nuclear pore complex and C9ORF72 long isoform is localized in the cytoplasm 83. Consistent with this prediction, C9ORF72 has been reported to associate with Rab proteins and thus involved in the regulation of endocytosis and autophagy 17. Earlier expression study in mouse showed C9ORF72 is highly expressed in neuronal cells 73. However, recent studies have observed highest levels of C9ORF72 expression in microglia and C9ORF72 knock out leads to an altered auto‐immune response with age related neuroinflammation leading to progressive splenomegaly and lymphadenopathy in mice 55.

Hypothesis of C9ORF72 haploinsufficiency leading to neurodegeneration was explored based on the observation of reduced C9ORF72 transcripts (V1, V2, V3) in patient tissues and iPSC‐derived neurons 1, 12, 16. Reduced expression of C9ORF72 is attributed to CpG hypermethylaion of promoter 37 and repeat expansion mutation binding to trimethylated H3K9 and H3K27 leading to epigenetic silencing 4. A number of *in‐vitro* studies using mouse primary neurons have shown that C9ORF72 interacts with Rab proteins and loss of C9ORF72 increases levels of p62 and positively regulates autophagy in the spleen and liver 72, 79. Interestingly, studies have observed increased level of p62, an autophagy marker, in post‐mortem tissues and motor neurons derived from C9ORF72 iPSC. Conversely, treatment with chloroquine (autophagy inducer) increased the vulnerability of iPSC‐derived C9ORF72 motor neurons to autophagy 1. Recent study in mouse primary motor neurons and human iPSC‐derived neurons showed C9ORF72 regulates cytoskeletal actin dynamics through phospho‐cofilin and shRNA mediated knockdown of C9ORF72 leads to axon outgrowth deficits 67. However, the absence of C9ORF72 loss of function (LOF) mutations in patient populations 28 along with rare C9ORF72 homozygous patients displaying a comparable clinical profile to heterozygous patients further argues against haploinsufficiency as the primary mechanism of disease 20.

### Pathological hallmarks of *C9ORF72^RE^*

One of the pathological signatures of *C9ORF72^RE^* mutation is the presence of RNA foci in cerebellum, motor cortex, spinal cord and hippocampus. These are expressed in both neurons and glial cells. RNA foci are generated by bi‐directional transcription of G_4_C_2_ repeat expansion (both sense and antisense) 2, 9, 12, 45. RNA foci are also observed in cortical neurons, motor neurons, astrocytes and oligodendrocytes derived from *C9ORF72^RE^* iPSCs 1, 16, 39, 44, 62. It is widely believed that G_4_C_2_ RNA foci sequesters RNA binding proteins and thereby causes toxicity to neurons and glial cells. A number of unbiased studies using co‐localization and immunoprecipitation have identified ribonucleoproteins, such as hnRNPA1, hnRNPA2/B1, hnRNPA3, ADARB2, Pur‐alpha, RanGAP1 that are associated with RNA foci 10, 16, 48.

Albeit rare, hairpin formation of repeat expansion mutations can trigger translation of the repeats through novel non‐ATG initiated (RAN) translation. Translation of such repeats are characterized in other diseases such as CTG expansion in 3′UTR of *DMPK* gene causing myotonic dystrophy type 1 (DM1) 86, and (CTG)n expansion in *ATX8* gene lead to bidirectional expression of poly‐Q inclusions causing spinocerebellar ataxia type 8 (SCA8) 50. Similarly, *C9ORF72^RE^* has been observed to be translated through an unconventional mechanism of repeat associated non‐ATG (RAN) translation 2, 49. RAN translation from both sense and antisense sequence leads to generation of five distinct di‐peptide repeats (DPR): poly‐GA, poly‐GP, poly‐GR, poly‐PR, poly‐PA 47. These DPR's are associated with p62^+ve^, ubiquitin^+ve^, TDP43^−ve^ cytoplasmic inclusions in both neurons and glia, a unique pathology of *C9ORF72^RE^* post‐mortem tissues 2, 49. Moreover, a recent transgenic mouse model of C9ORF72, where human C9ORF72 locus containing repeat expansion mutation is inserted in mouse, shows both RNA foci and DPRs in neurons and astrocytes thus recapitulating the pathology observed in patients 54.

### Toxic gain of function hypothesis

Expression of RNA foci and DPR's in various disease models of C9ORF72 suggests that toxic gain of function causes *C9ORF72^RE^* mediated ALS. However, it is still debatable whether sequestering ribonucleoproteins by RNA foci and/or DPR's causes neurotoxicity. Ectopic expression of G_4_C_2_ repeats in mouse primary neurons recapitulated RNA foci and correlated with apoptosis 33. Adeno viral mediated overexpression (G_4_C_2_)_66_ in mice resulted in expression of RNA foci, DPRs and p‐TDP43 inclusions. Furthermore, they were associated with loss of cortical neuron and significant motor deficits 7. These studies suggest a toxic gain‐of‐function mechanism of neurodegeneration. However, it is difficult to tease apart if RNA foci and/or DPR is causing neurotoxicity since both are expressed. Overexpression of poly‐PR DPR in an ATG‐dependent translation in mouse primary neurons and human iPSC‐derived motor neurons resulted in neurotoxicity as assayed by longitudinal survival analysis 80. Another study shows ectopic expression of poly‐GA in mouse cortical neurons is associated with reduced dendritic branching and increased caspase 3 mediated apoptosis 42. The majority of these studies overexpress G_4_C_2_ repeats in different models, however, they do not evaluate the consequence of physiological expression of *C9ORF72^RE^*. Human iPSC model systems addresses this issue. Some of the phenotypes observed in *C9ORF72* mutant motor neurons such as glutamate induced excitotoxicity 16, hypoexcitability 62 are mitigated by antisense oligonucleotide (ASO) treatment hinting that sequestering of ribonucleoproteins by RNA foci is a driver for disease mechanism. However, it is imperative to generate specific antibodies to inhibit DPRs to identify if DPRs are also contributing to the disease phenotype.

### Spread of DPRs

Among the di‐peptide repeats, poly(GA) is prone to aggregation *in‐vitro* and form cytoplasmic inclusions 6. Recently there has been emerging interest in cell to cell transmission of DPR's. Intercellular DPR seeding and transmission has been observed between neuron to neuron and neuron to glial cells 81, 85. Moreover, direct cell to cell interaction is not required for transmission noting that conditioned media and exosomes fractions are sufficient to induce transmission. These studies are performed predominantly using ectopic expression of DPRs under a strong promoter in various cell lines and primary neurons. However, this could lead to high expression and thus not representative of normal pathophysiology. To overcome this problem, Westergard *et al*, performed similar studies using co‐culture of iPSC‐derived C9ORF72 mutant sMNs and GFP labelled wildtype MNs. They observed transmission of poly‐GA, poly‐GP and poly‐GR DPRs thereby confirming its relevance at physiological levels 81. Further studies need to be performed to see if DPRs are detected in cerebrospinal fluid of patients which could be potentially used as a biomarker and also potentially an opportunity to neutralize pathogenic DPRs using antibody therapy.

### Cellular pathways that are disrupted in *C9ORF72^RE^* mediated ALS

#### Nuclear cytoplasmic protein shuttling defects

In a quest to elucidate the molecular mechanism for pathogenicity of hexanucleotide repeat expansion in C9ORF72, interactome and modifier studies revealed dysfunctional nuclear‐cytoplasmic trafficking of both proteins and RNA. Zhang *et al*, identified RanGAP1, a protein vital in shuttling of proteins containing nuclear localization signal, interacting with the G_4_C_2_ RNA. Ran GAP1 overexpression was reverted the ommatidial defects in the eye and restore locomotion deficits of *Drosophila* model expressing 30 G_4_C_2_ repeats. iPSC‐derived *C9ORF72^RE^* mutant motor neurons displayed mislocalization of RanGAP1 protein in cytoplasm and exhibited deficits in nuclear import of protein, which includes TDP43. Targeting the RNA foci by anti‐sense oligonucleotides (ASO) and facilitating nuclear import with a compound KPT 276, an inhibitor of exportin, reversed the phenotype observed in *Drosophila* model and human neurons 84.

Another independent study undertook an unbiased loss of function (LOF) screen to identify genes that modify G_4_C_2_ hexanucleotide repeat expansion mediated rough eye phenotype in *Drosophila* model 21. Eighteen modifiers that target the nuclear pore complex were identified. These included Nup50 and Nup153 RAN were identified to enhance the rough eye phenotype. ALYREF, a RNA binding protein responsible for RNA export, was identified to suppress rough eye phenotype. Consistent with these findings, iPSC‐derived *C9ORF72^RE^* motor neurons showed increased nuclear RNA retention when compared with controls 21. These studies show a toxic gain of function of either G4C2 RNA and DPRs alter the nuclear‐cytoplasmic transport and lead to pathogenicity.

### Vulnerability to ER and oxidative stress

Endoplasmic reticulum (ER) is an organelle necessary for production, folding and processing of secretory and transmembrane proteins. ER homeostasis, a balance between protein load and capacity to fold this load, is essential for proper folding and thus function of proteins. ER homeostasis is known to be perturbed by accumulation of misfolded mutant protein in the ER lumen. Recent studies have shown evidence ER stress is perturbed in *C9ORF72^RE^*. Dafinca *et al*, 11 showed that iPSC‐derived *C9ORF72^RE^* motor neurons exhibited elevated levels of Ca^2+^ in ER with increased levels of GRP78/BiP – a marker for UPR and ER stress response. Another independent study showed that iPSC‐derived *C9ORF72^RE^* motor neurons are vulnerable to tunicamycin, inducer of ER stress, in a dose dependent manner 26. These studies imply that protein homeostasis is perturbed in *C9ORF72^RE^* motor neurons, however causality for the ER stress remains unclear.

Oxidative stress is the result of an imbalance in the generation of free radicals and ability of the cell to neutralize/scavenge. The imbalance generally arises due to leakage of electrons from the mitochondria respiratory chain. Many studies, aside from identification of mutation in super oxide dismutase gene (SOD1), have shown evidence for increased oxidative stress in post mortem tissues of ALS patients. Increased levels of 8‐hydroxy‐2′‐deoxyguanosine (8‐OHdG), a marker for oxidized DNA, was observed in spinal cord ventral horn motor neurons 19. Recent study observed an age dependent (in culture) increase in DNA damage as measured by expression of γH2AX in iPSC‐derived *C9ORF72^RE^* motor neurons 40. Interestingly, ectopic expression of poly‐GR_80_ but not poly‐GA_80_ induced oxidative stress in *C9ORF72^RE^* motor neurons. Treatment of mutant motor neurons with Trolox, Vitamin E analog, marginally reduced oxidative stress suggesting oxidative stress may also contribute to the disease mechanism 40.

### Neuronal Excitability

Evidence for neuronal hyperexcitability has been broadly observed across the genetic spectrum of ALS and has been suggested to manifest pre‐symptomatically before potentially contributing to the degenerative process 3, 77. Emerging clinical functional data for *symptomatic* ALS patients that carry the *C9ORF72^RE^* shows axonal hyperexcitability in lower motor neurons 23 and the cortex 24, 65, 82. In direct contrast, cortical hyperexcitability has not been observed in *FTD* and *ALS‐FTD* patients, or *asymptomatic* patients, carrying *C9ORF72^RE^*s 65.

Neuronal excitability maybe influenced by intrinsic (isolated neuronal firing, functional receptor/ion channel expression) and/or extrinsic (synaptic and extra‐synaptic activity, connectivity) factors. Mechanistic studies are required to elucidate the full contribution of either to any potential excitability impairment in ALS. Electrophysiological approaches have therefore been applied to determine whether such functional impairments manifest at single neuron, synaptic and network levels in *C9ORF72^RE^* patient iPSC‐derived neurons.

Using multi‐electrode arrays, Wainger *et al*, 2014 showed network hyperexcitability in the form of increased basal spontaneous spike firing frequency in *C9ORF72^RE^* patient hiPSC‐derived cultures (14 days old, grown in co‐culture with primary mouse cortical astrocytes). Cultures in this study contained a low percentage (<20%) of Islet1^+^ motor neurons, but ultimately equivalent network hyperexcitability was observed in enriched mutant SOD1 patient iPSC‐derived HB9^+^‐GFP motor neuron cultures suggesting a convergence of an ALS hyperexcitable phenotype in lower motor neurons. Interestingly, patch‐clamp electrophysiological recordings from mutant SOD1 neurons determined a decreased functional expression in delayed‐rectifier potassium channels, but not sodium channels. Such channels have sustained activation kinetics and loss of functional expression is mechanistically consistent with increased capacity of neurons to fire action potentials and display an increased network firing frequency. Loss of functional expression of potassium channels with sustained kinetics is theoretically predicted to be a component in the generation of the observed hyperexcitability in symptomatic *C9ORF72^RE^* patient lower motor neuron axons 23. Retigabine, a Kv 7 delayed‐rectifier potassium channel family activator, has therefore been raised as a potential therapeutic in ALS 78.

In direct contrast, Sareen *et al* demonstrate that *C9ORF72^RE^* patient iPSC‐derived neuronal cultures (66–79 days old) containing SMI32^+^ motor neurons (∼60% enrichment) show intrinsic hypoexcitability with a decrease in the ability to fire action potentials in response to depolarization. Concurrently performed RNA‐sequencing indicated an increased expression of the delayed rectifier potassium channel, KCNQ3, which is mechanistically consistent with increased intrinsic hypoexcitability 62.

These divergent findings appear to be reconciled by temporal analysis of excitability of enriched MNs generated from *C9ORF72^RE^* patient iPSCs using patch‐clamp electrophysiology 14. Evidence for intrinsic hyperexcitability was found at an early time point (21–28 days) in culture in a subset of cells when classified according to their firing state. Recordings made from cells maintained up to 70 days showed that cells lost the capacity to fire action potentials and accordingly lost functional expression of sodium and potassium channel‐mediated conductances.

Together this suggests for *C9ORF72^RE^*–mediated ALS that a potential early intrinsic hyperexcitability potentially transitions, with time, into a hypoexcitable phenotype in motor neurons. However, the general contribution of intrinsic hyperexcitability to ALS has been further challenged by studies where data from other iPSC‐derived motor neuron models of ALS 38, 53 and the mutant SOD1 mouse 5, 13, 35 identify intrinsic hypoexcitability rather than hyperexcitability as the key physiological phenotype. Indeed, pharmacological interventions that increase excitability to improve motor neuron function and survival have been proposed 53, 64.

Differences in neuronal excitability may also arise through altered functional synaptic properties. Patch‐clamp recordings from *C9ORF72^RE^* patient iPSC‐derived cultures containing motor neurons are consistent with hypoexcitability where a reduced detection of cells with post‐synaptic events was observed after 7–10 weeks in culture 14. Such data is consistent with synaptic loss/reduced synaptic function observed in ALS patients and other models of ALS 35, 53, 63, 84. The pathological loss of synaptic input is supported by increasing evidence that RNA‐seq‐based studies on *C9ORF72^RE^* iPSC‐derived MN cultures and post mortem brain samples that consistently indicate altered expression of synaptically associated proteins 58, 62 and furthermore that C9ORF72 protein is observed to accumulate at the synapse 69. No other functional data is currently available that assesses synaptic physiology in *C9ORF72^RE^* models.

Subtle but important differences in experimental approaches likely accounts for the differences in excitability findings. These include potential for variability in iPSC‐derived cellular composition, including specific motor neuron identity, and maturation of iPSC‐derived cultures (for in‐depth review 60). For example, motor neuron cultures obtained from different protocols may include different levels of astrocytes that will also harbor *C9ORF72^RE^*. *C9ORF72^RE^*‐iPSC‐derived astrocytes have previously been shown to be toxic to iPSC‐derived motor neurons 44. The contribution of iPSC‐derived astrocytes to the observed intrinsic excitability of *C9ORF72^RE^*‐patient‐derived neurons remains unexplored. Furthermore, consideration of the culture composition has important implications for investigating synaptic physiology – it is unknown to the extent to which the synaptic connectivity in such cultures represents that of native circuits 60. In this regard, altered intrinsic neuronal excitability may simply reflect a homeostatic response to the extent of synaptic innervation within the culture 43.

The current evidence for hyper‐ vs. hypo‐excitability in ALS is clearly heterogeneous. The volume of evidence from *in vitro* models of ALS is weighted towards hypoexcitability in *C9ORF72^RE^* ALS, but this appears at clear odds with the existing hyperexcitability data obtained from symptomatic ALS *C9ORF72^RE^* patients. To move forward in understanding neuronal/circuit dysfunction in *C9ORF72^RE^*‐mediated ALS and FTD we must understand better the physiological representation of the iPSC‐derived neurons to those of native populations. In alliance with this, longitudinal clinical *C9ORF72^RE^* patient data must be obtained to verify and appropriately align observations made in iPSC‐derived studies 25.

### Excitotoxicity

Persistent glutamate‐mediated excitotoxicity is thought to play an important role in the progression of ALS including *C9ORF72^RE^* patients 8, 76. The development of the excitotoxicity hypothesis is multifaceted and has largely been developed from studies on mutant SOD1 and post mortem studies. This includes (1) the reduction of astroglial glutamate transporter expression that acts to reduce the clearance of synaptic glutamate; (2) an increase in functional Ca^2+^‐permeable glutamate‐gated AMPA receptor expression upon motor neurons and (3) a potentially reduced intracellular buffering Ca^2+^‐capacity leading to toxic elevations of intracellular Ca^2+^ 8, 76. It is thought that riluzole, the only licensed drug for ALS, largely acts through multiple mechanisms to reduce excitotoxicity 8. To date, only a few iPSC‐based studies have provided data to support the role of glutamate receptor‐mediated excitotoxicity in motor neurons.

Donnelly *et al*, 2013 demonstrated that cultures containing *C9ORF72^RE^* patient iPSC‐derived motor neurons (30%–40% HB9^+^) are highly susceptible to glutamate excitotoxicity, which is blocked by pharmacological inhibition of AMPA receptors, NMDA receptors and voltage‐gated Ca^2+^ channels. In association with a reduction in RNA foci, treatment of motor neurons with C9ORF72^RE^ anti‐sense oligonucleotides partially reduced the vulnerability to excitotoxicity suggesting the glutamate‐mediated excitotoxicity is associated with RNA toxicity. Interestingly, this study also observed nuclear co‐localization of ADAR2B2 RNA with RNA foci. This enzyme controls the post‐transcriptional modification of the GluA2 subunits (glutamine to arginine) that imparts Ca^2+^‐impermeability to GluA2‐containing AMPA receptors. Previously inefficient editing of the GluA2 subunit has been implicated shown in the post mortem spinal cord of sporadic ALS patients 32. The link between Ca^2+^‐permeable AMPA receptor‐mediated excitotoxicity in ALS and *C9ORF72^RE^* remains to be definitively determined.

In contrast, cortical neurons derived from *C9ORF72^RE^* FTD patients show altered AMPAR transcript regulation that is consistent with more functional Ca^2+^‐impermeable AMPA receptors 22. Furthermore, the functional AMPA receptor composition in oligodendrocytes derived from *C9ORF72^RE^* ALS patient iPSCs is predominantly Ca^2+^‐impermeable 39. These data indicate that excitotoxicity in these cell types is highly unlikely to be mediated via altered AMPA receptor composition. Importantly, this data is suggestive of both region and cell‐type specific *C9ORF72^RE^*‐mediated pathology.

### Glial Cross‐Talk

It is well recognized that non‐neuronal cellular phenotypes are likely to influence ALS/FTD disease progression beyond that of the astrocytic contribution to glutamate‐mediated excitotoxicity 29. Meyer *et al*, 2014 demonstrated that *C9ORF72^RE^*‐derived astrocytes were toxic to iPSC‐derived HB9^+^ motor neurons. Importantly, equivalent experiments using mutant SOD1 (human or rodent) derived astrocytes demonstrate a similar feature 15, 27, 41. Mutant TDP‐43 iPSC‐derived astrocytes appear not to be associated with toxicity to motor neurons 66 although mutant astrocytes in a TDP‐43 rat model do adversely influence motor neuron survival 75. It therefore remains unclear if astrocytes in ALS generally have an associated toxicity to motor neurons.

Recently, oligodendrocyte abnormalities have also been identified in experimental models and pathological studies of ALS 30. Rodent mutant SOD1 oligodendrocytes have impairments in differentiation from progenitor cells leading to insufficient re‐myelination of motor neurons and those that differentiate appear to exhibit morphological abnormalities consistent with impairments in maturation 30, 56. Mutant SOD1 oligodendrocytes that myelinate appear less able to metabolically support motor neuron axons 34. Furthermore, co‐cultures of mutant SOD1 patient iPSC‐derived oligodendrocytes were shown to impart toxicity to motor neurons 18. However, data emerging from *C9ORF72^RE^* patient iPSC‐derived oligodendrocyte studies are highly divergent with mutant SOD1 studies. Despite presenting pathological signatures of *C9ORF72^RE^*‐mediated disease, including RNA foci, *C9ORF72^RE^* patient iPSC‐derived oligodendrocytes were shown not to impart toxicity to motor neurons when maintained in co‐culture 18 and furthermore were not associated with any maturational or morphological impairment 39.

The analysis of factors (contact and conditioned media) regulating glial toxicity to motor neurons remains to be addressed although early studies have identified promising potential therapeutic candidates 70. Moreover, the very recent ability to generate functional microglia from human pluripotent cultures is of great interest for ALS especially for *C9ORF72^RE^*‐mediated disease that is maximally expressed in microglia 51, 55.

### Summary

iPSC technology allows the generation of multiple *in vitro* cellular types that are relevant to modeling *C9ORF72^RE^*‐mediated disease. Critically, patient‐derived material largely recapitulates major *C9ORF72^RE^* pathology and beyond this has been used to uncover new potential disease hypotheses. Data from a range of *C9ORF72^RE^* models will ultimately be required to reinforce these ideas. In this regard, it is important to fully understand the nature of the iPSC‐derived material and how it reflects that of native *in vivo* disease progression to place such results into appropriate context. Nonetheless iPSC technology will continue to be an important platform to explore *C9ORF72^RE^*‐mediated disease. The increasing ability of these systems to be adapted and scaled for high‐throughput screening for novel pharmacological compounds is of particular promise.
